# To Sleep Perchance to Dream - Not; Nyctophobia From COVID-19 Induced Hyposmia

**DOI:** 10.1192/j.eurpsy.2023.1709

**Published:** 2023-07-19

**Authors:** S. Kalita, D. Birwatkar, R. Cosme, A. Hirsch

**Affiliations:** 1 Psychiatry; 2 Spartan Health Sciences University, Vieux Fort, Saint Lucia; 3 Psychiatry, Rush University Medical Center; 4 Psychiatry, Smell and Taste Treatment & Research Foundation, Chicago, United States

## Abstract

**Introduction:**

Fear of sleep (nyctophobia), has been attributed to myriad conditions ranging from benign nocturnal panic attacks and Morvan’s syndrome (Ekambaram, 2021). Positional dependent hyposmia as an origin of nyctophobia has not heretofore been described.

**Objectives:**

Increase awareness for correlation between nyctophobia and hyposmia in individuals with COVID-19.

**Methods:**

This 52-year-old woman presented with sudden onset of loss of smell and taste with COVID-19, which returned to 85% of normal. Five months prior to presentation she was reinfected with COVID-19, and her smell and taste dropped to 20-50% of normal which improved. However, her symptoms worsened when she would lie down, to 30% of normal, but would improve with standing, moving and sitting. Even reclining for a short nap caused her sense of smell to drop, requiring her to stand for hours before her sense of smell would return leading to her nyctophobia that forced her to move around all the time. She altered her lifestyle and assiduously avoided lying down. When so overwhelmed by tiredness she would sleep sitting up. Prior to her chemosensory problems she slept well without any fear of lying down or sleeping.

**Results:**

Psychiatric exam: speech: coherent, relevant without circumstantiality, normal pace and volume. Mood: normal. Oriented x 3. Able to remember 7 digits forward and 5 backwards. Able to recall 3/4 objects without reinforcement. Interpretation of similarities: normal. Proverbs: normal. Calculation: normal. Neuropsychiatric testing: Clock Drawing Test: 4/4 (normal). Animal Fluency Test: 22 (normal).

**Conclusions:**

Nyctophobia, fear of positional dependent loss of smell, highlights the importance of smell to narcissistic perception of self. More than just one of senses, olfaction is important for mood regulation, memories and quality of life. Associated with chemosensory dysfunction, this is associated with 96% incidence of DSM-IIIR Axis I or II diagnoses, with the most common Axis I diagnosis being generalized anxiety disorder and dysthymia (Hirsch, 1996). This can be understood that the olfactory lobe is anatomically part of limbic system (MacLean, 1973). Smell fascilitates socialisation as well as maintaining interelationships; sexual dysfunctioning is seen in 17% of the population with olfactory loss (Hirsch, 1998). Deposition of memory engrams are facilitated as manifest with olfactory evoked nostalgia seen in 84% of the general population (Hirsch, 1992). Quality of life is reduced in 68% of patients who demonstrate smell loss (Deems, 1991). The importance of smell is thus intrinsic to an individual’s wellbeing, and the threat of loss of such sensation with change in position with sleep, was the precipitating event leading to nyctophobia in this subject. Assessing for fear of loss of smell in those with nyctophobia, especially with past COVID-19 infection, allows for increased understanding of etiology and indicates potential treatment approaches.

**Disclosure of Interest:**

None Declared

